# A Dermoid Cyst

**Published:** 1888-08

**Authors:** John Preston

**Affiliations:** Assistant Physician at the State Lunatic Asylum, Austin, Texas


### A DERMOID CYST.

By John Preston, M. D.,

*Assistant Physician at the State Lunatic Asylum, Austin, Texas.*

For Daniel's Texas Medical Journal.

MRS. JANE T—— was admitted into the State Lunatic Asylum September 4, 1884, and was then 45 years of age. She had been insane for nine years previous to her admission. She was classed as a case of chronic dementia. During her residence in the asylum she was usually quiet and orderly and gave little trouble in her management. She was a victim of mild delusion, imagining at times that she had a clock in her stomach which gave her much trouble.

There is no evidence of any mental improvement in her case during her stay at the asylum.

Her physical health was generally good, though she frequently complained, during the last year of her life, of pain in the epigastric region. A mild purgative generally gave relief.

On July 16, 1888, she became afflicted with cramps in her bowels, which were very severe from the beginning, and resembled the pains of cramp colic. Hypodermic injections of morphia were used freely, and external applications of mustard and turpentine stupes. Enemas of warm water and soap were also given repeatedly, but without benefit, except temporarily. Her pains would recur in a short time with greater severity, and seemed unbearable. Her suffering continued almost without intermission, until 4.30 a. m., July 17, 1888, when she died.

An autopsy was held five hours after death. Body was found well nourished, very fleshy; stomach healthy; a stricture was discovered in the small intestine, causing an obstruction of the bowel. Several feet of the intestine above the seat of constriction were very much congested and very dark in appearance, and in a short time would have become gangrenous from obstruction of the circulation. The stricture was evidently the cause of her excruciating pain and of her death.

The large intestine was apparently in a healthy state, nothing unnatural was found in the condition of any of the other abdominal organs. The uterus was small, though normal in appearance, ovaries normal, bladder healthy. A dermoid cyst, pear shaped, four inches in length and three inches at its base, was discovered in the right iliac region, attached by a short pedicle to the broad ligament. With the exception of this attachment it floated freely above the pelvis, there being but slight adhesions. Upon opening the tumor it was found to contain a matted mass of hair of yellowish brown color. It grew from a dermoid structure, at the upper or large end of the capsule enveloping it. This was much thicker than any other part of the cyst, and presented the exact appearance of true skin of the scalp. The inner surface of the cyst was covered with short hair and a calcareous deposit. The capsule was distended with an oily substance, resembling an emulsion of linseed oil. The membrane, which was fibrous, was very tough, and in shape and consistence not unlike a distended bladder.

Dermoid cysts have been found in almost every part of the body. They contain hair, teeth, bone, cartilage and an oily or cheesy substance. They are very curious, and hard to explain as to their origin and cause.

Authors differs widely in their theories accounting for these wonderful tumors. One says, "they are due to an imperfectly developed ovum being included in the body of another which arrives at maturity. "Inclusion of abnormal structures, where there is a dipping in of the epiblast to meet the hypoblast in fœtal life and the pinching off of the same." "Hyperecchesis—which means that the ovum has within its origin buds of certain tissues which go on to rudimental formation of these tissues, without a fusion with the male germ," is the view of another.

The old idea that they are due to twin pregnancies, one fœtus dying in early fœtal life and all or part of its debris entering into the other and there becoming encysted and organized, is probably the most plausible theory. Much has been written upon this subject, but so far little light has been thrown upon it. The origin and cause of dermoid cysts still remain a mystery, and probably always will.

